# Ultrasonographic measurement of masseter muscle thickness and elasticity in patients with symptomatic apical periodontitis

**DOI:** 10.1007/s00784-026-06928-2

**Published:** 2026-05-22

**Authors:** Nezif Çelik, Mehmet Emin Dogan

**Affiliations:** 1 Faculty of Dentistry, Department of Endodontics, Harran University, Sanlıurfa, 63300 Turkey; 2 Faculty of Dentistry, Department of Dentomaxillofacial Radiology, Harran University, Sanlıurfa, 63300 Turkey

**Keywords:** Symptomatic apical periodontitis, Masseter muscle, Shear wave elastography, Ultrasonography, Endodontic treatment

## Abstract

**Objective:**

The purpose of current study was to evaluate changes in masseter muscle thickness (MMT) and elasticity in patients with symptomatic apical periodontitis (SAP) using ultrasonography before and after endodontic treatment.

**Methods:**

This prospective controlled clinical study inclusive 30 patients diagnosed with SAP and 30 systemically and orally healthy individuals. MMT was assessed using B-mode ultrasonography, and tissue stiffness was measured with shear wave elastography (SWE). Measurements were obtained bilaterally at rest and during maximum clenching. In the case group, the second measurement was performed 1-month after the completion of root canal treatment and without the observation of symptoms. Data were investigated using independent and paired samples t-tests, statistical importance level of *P* < 0.05 was accepted.

**Results:**

Intra-observer agreement was good (κ = 0.83). At baseline, the right MMT was importantly lower in the case group compared with the control group, both at rest and during clenching (*p* = 0.003 and *p* = 0.020, respectively). Similarly, right-sided SWE values obtained during clenching were significantly reduced in the case group (*p* = 0.003), whereas no significant difference was found for resting SWE measurements.

On the left side, resting measurements did not diverge importantly between groups. However, during clenching, both muscle thickness and SWE values were significantly lower in the case group (*p* = 0.024 and *p* = 0.001).

**Conclusion:**

SAP may cause alterations in MMT and stiffness. The improvement observed in ultrasonographic parameters after root canal treatment suggests that these changes may represent a reversible functional adaptation to acute periapical inflammation.

**Clinical relevance:**

The clinical significance of this study focuses on elucidating how SAP and associated pain can lead to secondary adaptations in the masseter muscle, such as protective co-contraction or muscle protection.

## Introduction

Apical periodontitis (AP) is an inflammatory situation of the periapical tissues that arises from microbial invasion of the root canal system and results in progressive destruction of the surrounding apical structures [1]. AP may present in different clinical and histopathological forms. These clinical presentations encompass symptomatic apical periodontitis (SAP), acute apical abscess, asymptomatic AP, and chronic apical abscess [2]. SAP is a condition in which the acute inflammatory response developing in the periapical tissues is clinically prominent. In SAP, increased vascular permeability and the release of inflammatory mediators and pro-inflammatory cytokines lead to edema within the periodontal ligament; this results in the tooth being perceived as slightly extruded within the alveolus and causes pain during function. Radiographic findings are often minimal or not yet evident; therefore, the diagnosis of SAP is primarily based on clinical findings. The main clinical feature in affected patients is severe, spontaneously developing pain, which is typically localized and throbbing in nature. Depending on the pulpal status, vitality tests may yield negative or delayed positive responses. In most cases, soft tissue swelling is not observed, and sensitivity to thermal stimuli is generally absent or reduced. In contrast, pain on biting and tenderness to percussion are characteristic clinical findings [3]. This pain is not merely a consequence of local tissue damage but represents a complex biological process shaped by nociceptor activation and neuro-immune interactions. During periapical inflammation, the release of mediators such as prostaglandins, bradykinin, calcitonin gene-related peptide, substance P and pro-inflammatory cytokines directly stimulates peripheral nociceptors, leading to the transmission of pain signals to the central nervous system via trigeminal nerve pathways. This process activates not only the peripheral nervous system but also the central nervous system, thereby enhancing the perception of acute orofacial pain [4, 5]. Owing to this neuroanatomical and neurophysiological relationship, dental pain and inflammation may not remain confined to the periapical tissues but may also induce secondary changes in functionally related musculoskeletal structures. In particular, the masseter muscle is one of the primary muscles responsible for masticatory function and the generation of occlusal force, and it is innervated by the trigeminal motor system [6]. Dental occlusion is highly sensitive to pain and inflammatory stimuli. The acute pain and neurogenic inflammation associated with SAP may contribute to functional and structural alterations in the masseter muscle through several interconnected mechanisms, including increased reflex muscle activity, changes in muscle tone regulation, disturbances in local microcirculation, and inflammation-related tissue edema [7]. These processes are thought to reflect adaptive or protective neuromuscular responses to dental pain rather than direct tissue injury [8]. However, despite being frequently observed in clinical practice, these potential effects have not yet been sufficiently demonstrated using objective imaging modalities.

The masseter muscle can be evaluated using imaging techniques such as ultrasonography (USG), magnetic resonance imaging (MRI), and computed tomography (CT) [8]. The use of USG in dentistry is becoming increasingly widespread due to its advantages, including the absence of ionizing radiation, non-invasiveness, ease of application, and good patient tolerance. Recent advances in ultrasonographic techniques have enabled the evaluation of not only the morphological characteristics of the muscle but also its mechanical and functional properties. In particular, these techniques provide indirect yet valuable information regarding muscle tone, activity, and inflammatory changes by assessing tissue stiffness and resistance to deformation [9, 10]. A previous ultrasound study reported that the thickness and stiffness of the masseter muscle changed due to unilaterally chewing [11].

The clinical relevance of this study is centered on elucidating how localized periapical inflammation and its associated pain can lead to secondary adaptations in the masticatory muscles, such as protective co-contraction or muscle guarding. Identifying these morphological and biomechanical alterations in the masseter muscle through objective and non-invasive methods, such as B-mode ultrasonography and shear wave elastography, provides clinicians with a deeper understanding of the extra-dental clinical manifestations of endodontic pathologies.

The hypotheses for this study were formulated as follows:

- Null Hypothesis (H0): There is no significant difference in MMT and stiffness between the presence of SAP and a healthy state, and root canal treatment does not produce any significant changes in these parameters.
- Primary Hypothesis (H1): Patients diagnosed with SAP exhibit significantly lower MMT and stiffness on the affected side compared to healthy individuals, and successful root canal treatment results in a statistically significant increase in these parameters.

Current study aims to assess MMT and elasticity using USG in patients with SAP, before and after root canal treatment (RCT), to evaluate the potential structural and functional effects of acute dental pain and inflammation. Although USG and shear wave elastography have been increasingly used to assessment masseter muscle morphology and mechanical properties in temporomandibular disorders and other inflammatory conditions, there is currently no published study specifically investigating MMT and elasticity in patients with SAP. In particular, ultrasonographic changes before and after RCT have not yet been clearly documented.

## Material and method

The protocol was established on the clinical trials platform. (http://www.clinicaltrials.gov, Identifier: NCT 06734559). This prospective, single-center controlled clinical trial received approval from the Clinical Research Ethics Committee of Harran University (decision number. HRÜ/24.15.22).

### Sample size

**Fig. 1:**
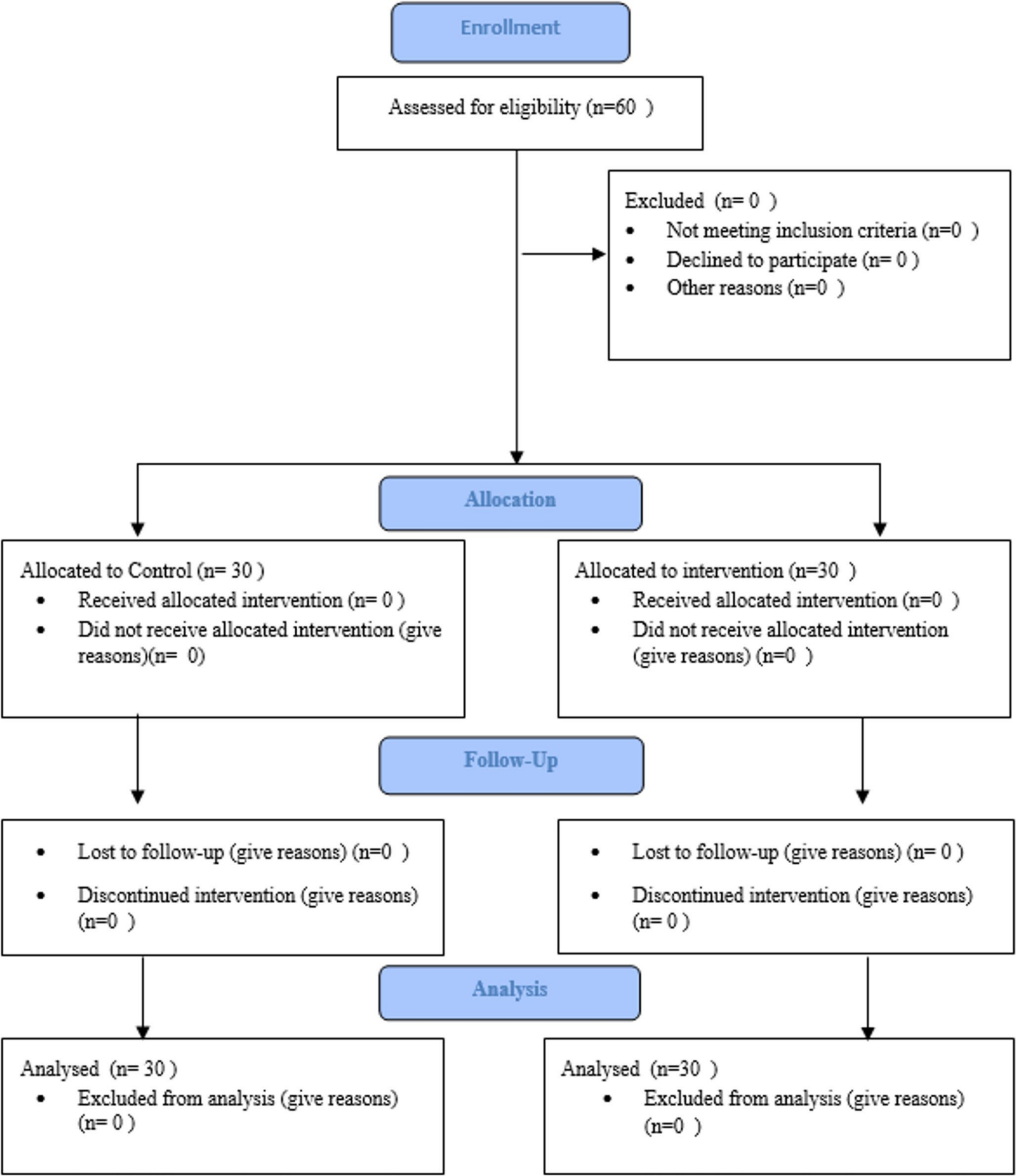
A flow diagram for the inclusion of participants in the study

G Power 3.1.9.7 (Heinrich-Heine-Universität Düsseldorf) was preferred for calculating sample size. For the paired sample t-test, it was determined that at least 27 participants were required when effect size d = 0.5, 80% test power (1-β = 0.80) and α = 0.05 were accepted. In this study, a total of 60 people were included, 30 cases and 30 controls. (Fig. 1).

### Eligibility criteria

Participants were recruited from patients presenting to the Department of Endodontics, Faculty of Dentistry, Harran University, between December 2024 and May 2025. All participants were informed about the study’s objectives, procedures, benefits, and potential risks before participating. Only those who signed an informed consent form were included.

Inclusion criteria:

- Systemically healthy adults aged 18–65 years (American Society of Anesthesiologists Class I).
- Diagnosis of acute apical periodontitis.
- Presence of at least four posterior occlusal units (one premolar pair counted as one unit; one molar pair counted as two units).
- Age ≥ 18 years.
- Absence of maxillofacial pathology or systemic disease.
- No history of temporomandibular joint or masticatory muscle disorders.
- No complaints of dysphoni.
- No diagnosed muscular disorders.

Exclusion criteria.

- Presence of systemic disease.
- Current use of any medication.
- Fewer than 20 natural teeth.
- Presence of prosthetic restorations (fixed dental prostheses, single crowns or removable prostheses).
- Dental implant restorations.
- History of periodontal treatment within the previous year.
- Diagnosis of symptomatic pulpitis.
- Pregnancy.
- Smoking.
- Unilateral edentulism.

### Treatment protocol

All RCTs were accomplished by a single operator in 2 visits. Local anesthesia was administered using 2 mL of 4% articaine with 1:100.000 epinephrine (Maxicaine Forte; Vem İlaç, Türkiye). Access cavity was prepared using a 014 diamond round bur under rubber dam isolation. Working long (WL) was determined using an electronic apex locator (Root ZX II; J. Morita Corp., Kyoto, Japan) in combination with a size 10 K-file. ProTaper Next X1 (#17, 0.04 taper) and X2 (#25, 0.06 taper) files (Dentsply Sirona, Ballaigues, Switzerland) were used sequentially with an endodontic motor (VDW Silver Reciproc; VDW, Munich, Germany) using gentle in-and-out movements in accordance with the manufacturer’s instructions. Following every third pecking motion, the instrument was file withdrawn and cleaned, and the canal was irrigated with 2 mL of 5.25% sodium hypochlorite (NaOCl) (Cerkamed, Stalowa Wola, Poland). Apical patency was re-established before reinstrumentation. This protocol was continued until the predetermined working length was achieved. This sequence was repeated until the WL was reached. Final irrigation was performed using 5 mL of 17% ethylenediaminetetraacetic acid (EDTA), followed by 2.5 mL of distilled water and 5.25% sodium hypochlorite (NaOCl). As an intracanal medication, calcium hydroxide was placed after the canals were dried with sterile paper tips, and the cavity was temporarily sealed with glass ionomer material for 14 days. At the end of the initial 14-day period, patients were recalled, the canals were re-irrigated, and calcium hydroxide dressing was renewed for an additional 14 days. Upon completion of the 28-day interval, ultrasonographic evaluations were repeated and the findings were documented. The 28-day intracanal medication protocol, involving two consecutive 14-day cycles of calcium hydroxide application, was employed as a standardized institutional procedure for treating cases of SAP.

### Case group

In this study, ultrasound imaging was performed by M.E.D., who has 7 years of experience at the Department of Dentomaxillofacial Radiology, Faculty of Dentistry, Harran University. A LOGIQ™ P9 XDClear (GE Ultrasound Korea) ultrasound machine and an L3-12-RS (3–12 MHz) extraoral linear probe were used for the procedures. This group included patients with SAP presenting with symptoms in one of the posterior teeth.

Resting measurements were performed in a quiet and comfortable environment, with the patient at rest in the supine position. During the measurements, participants were asked to keep their heads straight, avoid occluding their teeth, and refrain from swallowing. Measurements during clenching were obtained from images showing the maximum clenching position.

To obtain a good image, a standard water-based acoustic coupling gel (Aqua Sonic 100 Ultrasound Gel) was used; the probe was placed on the skin surface without applying pressure.

For standardization purposes, mode B was used, with a scan depth of 38 mm, frame rate of 21 fps, gain of 57, image map F/0 and dynamic range of 69. Measurements were taken at the thickest point of the masseter muscle.

**Fig. 2:**
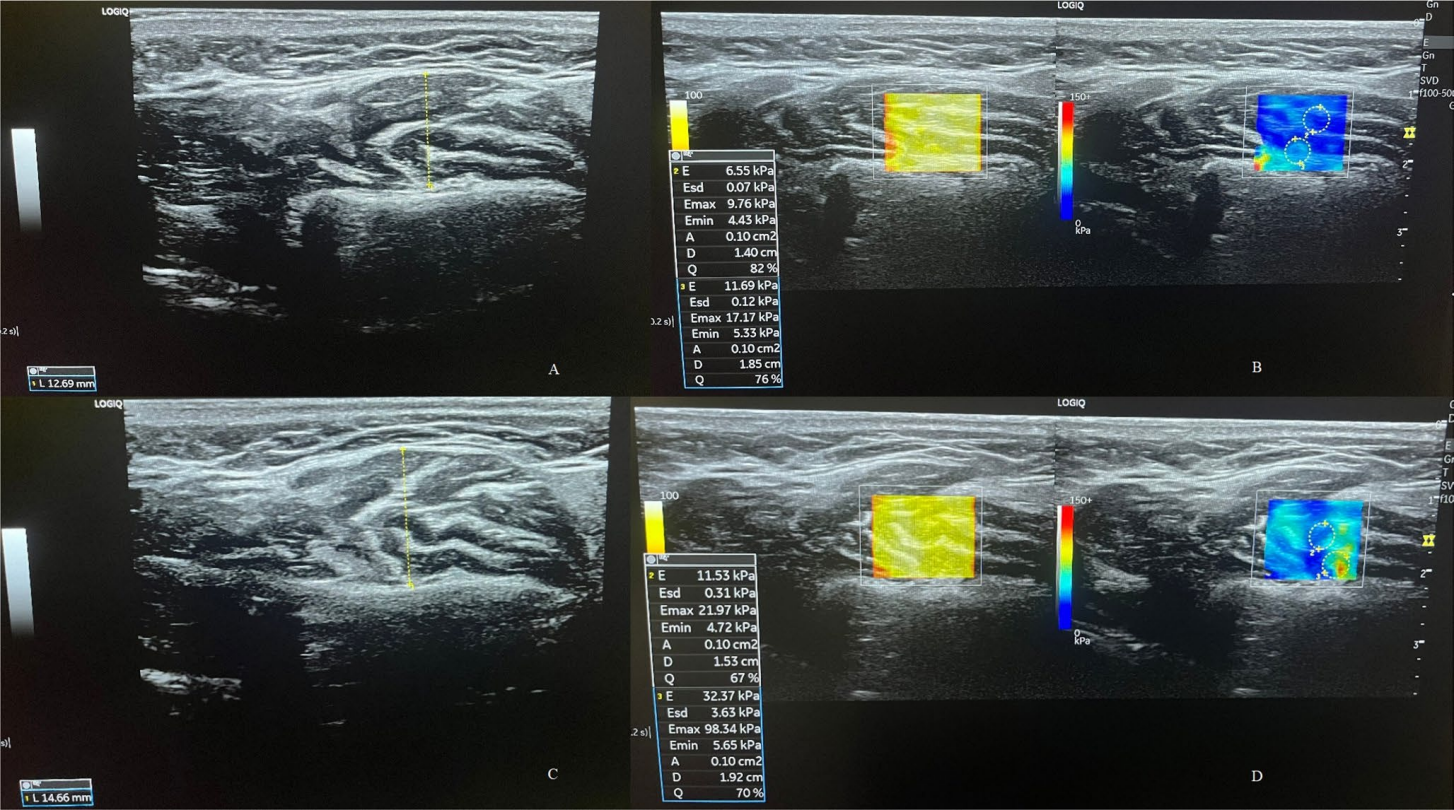
Resting MMT (**A**), Resting masseter muscle SWE (**B**), Clenching MMT (**C**), Clenching masseter muscle SWE (**D**)

For standardization purposes, elastography measurements were performed using shear wave elastography (SWE) mode with a scan depth of 38 mm, frame rate of 100–500 frames per second, gain of 75, image map SW1, and dynamic range 69. Three consecutive measurements were taken for each muscle, and the average of these measurements was recorded. The area of measurement (ROI) was set to 0.10 cm². The value saved as Young’s modulus was called the elasticity value and was expressed in kPa (Fig. 2).

In this study, stiffness measurements were taken from the area marked blue on the color map. The probe was placed parallel to the occlusal plane and perpendicular to the fibers of the masseter muscle, between the zygomatic arch and the lower edge of the mandible. The probe was moved over the muscle until the desired image was obtained, and thickness measurements were performed to determine the maximum distance between the medial and lateral fascia in the resulting image. Then, the SWE system was activated. The ROI area was set to 0.10 cm^2^. Elastography measurements were performed on the elastography image obtained with the SWE system activated. Measurements were taken in the case group as an initial measurement before treatment and a second measurement one month after the symptoms had subsided following treatment.

### Control group

A total of 30 healthy patients without any oral health problems were included in the study. Ultrasound measurements of the masseter muscle were taken on the first and 28 days, and the data were recorded. These data were then statistically compared to assess the changes in the masseter muscle, and the results were compared with the ultrasound group.

### Statistical analysis

SPSS Statistics v25 (Armonk, NY, USA) software was used for the statistical analysis of the data. The Shapiro-Wilk test was used to assess whether the data followed a normal distribution. Values such as number, percentage, minimum, maximum, and mean were calculated using descriptive statistics and frequency analysis. For group comparisons of continuous variables with normal distributions, the independent samples t-test was preferred. Levene test was used to determine homogeneity. The paired sample t-test was used for repeated measures analysis. The kappa test was applied to determine intra-observer agreement. The statistical impotance level was considered to be *p* < 0.05.

## Results

In this study, intra-observer agreement was at a good level, and the kappa value was found to be (0.83). The case group occurred of (14) 46.7% male and (16) 53.3% female. The control group consisted of (14) 46.7% female and (16) 53.3% male. The average age of the case group was 34.10 ± 14.16 years, and the average age of the control group was 32.56 ± 4.18 years. The Levene test result (*P* > 0.05) indicates a homogeneous distribution of variances across independent variables.

In the case group, the mean right masseter muscle diameter in the initial measurements taken at rest was 9.89 ± 1.17 mm, while in the control group, the right MMT was 11.17 ± 4.24 mm, both of which were higher than the case group. This difference was statistically significant (*P* = 0.003). At rest, the mean right masseter muscle SWE of the case group was 8.82 ± 3.08 kPa, while the right masseter muscle SWE of the control group was 9.50 ± 2.94 kPa, higher than the case group. This difference was not statistically significant (*P* = 0.743). In the case group, the mean thickness of the right masseter muscle clenching was 11.98 ± 2.27 mm in the initial measurements, while the mean thickness of the right masseter muscle clenching in the control group was 13.30 ± 3.26 mm, higher than the case group. This difference was found to be statistically significant (*P* = 0.020). The mean initial SWE measurement of the right masseter muscle in the case group during teeth clenching was 19.99 ± 6.48 kPa, while the mean SWE of the right masseter muscle in the control group during teeth clenching was 39.75 ± 13.35 kPa, which were detected to be higher than the case group. This difference was found to be statistically meaningful (*P* = 0.003). When comparing the mean resting left MMT and SWE measurements between the initial measurements of the case group and the control group, the control group was found to have higher left MMT and SWE values than the case group. This difference was not statistically significant (*P* > 0.05). When comparing the mean left masseter muscle diameter and SWE in the clenching state between the initial measurements of the case group and the control group, the control group’s left MMT and SWE values in the clenching state were detected to be higher than those of the case group. This differentiation was statistically meaningful (*P* = 0.024, *P* = 0.001) (Table 1).

**Table 1:** Mean masseter muscle thickness and stiffness measurements of the case group (first measurement) and the control group

	group	N	Mean ± Std.Deviation	*p*
Resting right masseter thickness	case	30	9.89 ± 1.17 mm	0.003*
	control	30	11.17 ± 4.24 mm	
Rest right masseter SWE	case	30	8.82 ± 3.08 kPa	0.743
	control	30	9.50 ± 2.94 kPa	
Clenching right masseter thickness	case	30	11.98 ± 2.27 mm	0.020*
	control	30	13.30 ± 3.26 mm	
Clenching right masseter SWE	case	30	19.99 ± 6.48 kPa	0.003*
	control	30	39.75 ± 13.35 kPa	
Resting left masseter thickness	case	30	9.85 ± 1.59 mm	0.122
	control	30	10.02 ± 2.53 mm	
Resting left masseter SWE	case	30	9.36 ± 3.24 kPa	0.129
	control	30	10.53 ± 2.60 kPa	
Clenching left masseter thickness	case	30	11.41 ± 1.88 mm	0.024*
	control	30	13.15 ± 3.65 mm	
Clenching right masseter SWE	case	30	20.84 ± 7.98 kPa	0.001*
	control	30	38.77 ± 13.94 kPa	

When the second measurements of the case group were examined, the mean left and right MMT and SWE at rest were compared between the case group and the control group. The control group had higher MMT and SWE values than the case group. This difference was not statistically significant (*P* > 0.05). When the second measurements of the case group were examined, the mean MMT of the right and left masseter muscles in the clenching state was compared between the case group and the control group. The MMT value of the control group was detected to be higher than that of the case group. This difference was not statistically significant (*P* > 0.05). However, in the second measurements, the SWE values during right and left masseter muscle contraction were found to be higher in the control group compared to the case group. This difference was observed to be statistically significant (*P* = 0.000) (Table 2). However, a significant increase compared to the initial value was observed in the case group.

When comparing the first and second measurements in the case group, considering the affected side, it was found that the right masseter muscle thick was higher in the second measurement at rest when the affected side was the left, but this difference was not statistically significant (*P* = 0.182). When the affected side was the left, it was observed that the left masseter muscle SWE at rest and the left MMT and SWE under compression were both higher in the second measurement than in the first measurement (*P* < 0.05). When the affected side was the right, the thickness and SWE values of the right masseter muscle were found to be significantly higher in both resting and contracted second measurements (*P* < 0.05). However, although the second measurements were higher than the first measurements on the left side, no significant difference was found (*P* > 0.05) (Table 3). No adverse situations were encountered during the study period.

**Table 2:** Mean masseter muscle thickness and stiffness measurements in the case group (second measurement) and the control group

	group	N	Mean ± Std.Deviation	*p*
Resting right masseter thickness	case	30	10.94 ± 1.75 mm	0.786
	control	30	11.17 ± 4.24 mm	
Resting right masseter SWE	case	30	10.44 ± 2.32 kPa	0.177
	control	30	9.50 ± 2.94 kPa	
Clenching right masseter thickness	case	30	12.85 ± 1.94 mm	0.527
	control	30	13.30 ± 3.26 mm	
Clenching right masseter SWE	case	30	26.59 ± 10.66 kPa	0.000*
	control	30	39.75 ± 13.35 kPa	
Resting left masseter thickness	case	30	10.19 ± 1.68 mm	0.764
	control	30	10.02 ± 2.53 mm	
Resting left masseter SWE	case	30	10.55 ± 2.62 kPa	0.973
	control	30	10.53 ± 2.60 kPa	
Clenching left masseter thickness	case	30	12.21 ± 2.42 mm	0.246
	control	30	13.15 ± 3.65 mm	
Clenching left masseter SWE	case	30	27.15 ± 9.50 kPa	0.000*
	control	30	38.77 ± 13.94 kPa	

**Table 3:** Mean first and second measurements of masseter muscle thickness and stiffness according to the diseased side

Diseased side				*N*	Mean ± Std. Deviation	*P*
Left	Resting right masseter thickness		First measurement	14	9.82 ± 1.37 mm	0.118
			Second measurement	14	10.76 ± 1.88 mm	
	Resting right masseter SWE		First measurement	14	9.13 ± 3.88 kPa	0.614
			Second measurement	14	9.69 ± 2.02 kPa	
	Clenching right masseter thickness		First measurement	14	12.34 ± 2.69 mm	0.185
			Second measurement	14	13.07 ± 1.59 mm	
	Clenching right masseter SWE		First measurement	14	21.24 ± 8.20 kPa	0.025*
			Second measurement	14	27.94 ± 13.28 kPa	
	Resting left masseter thickness		First measurement	14	9.51 ± 1.42 mm	0.182
			Second measurement	14	10.33 ± 1.63 mm	
	Resting left masseter SWE		First measurement	14	8.17 ± 2.51 kPa	0.000*
			Second measurement	14	10.67 ± 1.49 kPa	
	Clenching left masseter thickness		First measurement	14	10.94 ± 1.69 mm	0.001*
			Second measurement	14	12.05 ± 2.36 mm	
	Clenching left masseter SWE		First measurement	14	17.47 ± 4.86 kPa	0.008*
			Second measurement	14	24.14 ± 10.73 kPa	
Right	Resting right masseter thickness		First measurement	16	9.96 ± 1.01 mm	0.000*
			Second measurement	16	11.10 ± 1.67 mm	
	Resting right masseter SWE		First measurement	16	8.55 ± 2.27 kPa	0.001*
			Second measurement	16	11.09 ± 2.42 kPa	
	Clenching right masseter thickness		First measurement	16	11.66 ± 1.86 mm	0.000*
			Second measurement	16	12.66 ± 2.24 mm	
	Clenching right masseter SWE		First measurement	16	18.90 ± 4.50 kPa	0.001*
			Second measurement	16	25.41 ± 7.98 kPa	
	Resting left masseter thickness		First measurement	16	10.16 ± 1.71 mm	0.687
			Second measurement	16	10.07 ± 1.76 mm	
	Resting left masseter SWE		First measurement	16	10.40 ± 3.52 kPa	0.961
			Second measurement	16	10.45 ± 3.37 kPa	
	Clenching left masseter thickness		First measurement	16	11.81 ± 1.99 mm	0.139
			Second measurement	16	12.35 ± 2.53 mm	
	Clenching left masseter SWE		First measurement	16	23.78 ± 9.10 kPa	0.075
			Second measurement	16	29.78 ± 7.68 kPa	

*p < 0.05, std: standard

## Discussion

In this study, MMT and SWE values were examined before and after treatment in SAP patients using ultrasound. Our literature review revealed that no similar study has been conducted previously. Therefore, this study is clinically significant as it fills this gap in the literature.

USG is a reliable imaging method that allows for the non-invasive evaluation of the morphological and functional characteristics of the masseter muscle. In particular, SWE has been widely used in masseter muscle studies in recent years due to its ability to quantitatively measure the mechanical properties of muscle tissue [11]. The utilization of SWE in this study provides a more objective quantification of muscle behavior compared to traditional methods. Recent research indicates that masseter muscle stiffness typically exhibits a substantial increase approximately 67% when moving from a relaxed state to maximal contraction [12]. This significant physiological shift underscores the high sensitivity of SWE in detecting subtle pathological deviations in muscle tone [13]. By capturing these changes, the method allows for a precise longitudinal monitoring of the recovery process after the removal of the inflammatory stimulus [14].

Our findings indicate that acute inflammatory processes caused by periapical infection lead to significant changes in the masseter muscle. Specifically, in the initial measurements, the thickness of the right masseter muscle in the case group was found to be significantly lower than in the control group, both at rest and during clenching, while the SWE values during clenching were also significantly decreased. These results are consistent with previous literature showing that inflammatory conditions can affect the characteristics of the masticatory muscle [15, 16]. It is noteworthy that our findings regarding decreased functional stiffness in SAP patients contrast with some studies on chronic conditions like bruxism or myofascial pain, which often report hypertonicity or increased resting stiffness [17, 18]. This suggests that acute odontogenic pain triggers a unique protective suppression of muscle activity, rather than the chronic adaptation seen in muscle disorders [19]. Such a response aligns with the ‘Integrated Pain Adaptation Model,’ where the central nervous system reorganizes motor unit recruitment as a defensive mechanism to limit further tissue strain during the peak of acute inflammation [20, 21]. In the same conditions, although the right masseter muscle resting SWE values were higher in the case group than in the control group in the initial measurements, the difference was not statistically significant. These results are similar to studies showing that factors affecting chewing function also affect muscle stiffness [22].

On the left side, although muscle thickness and SWE values at rest were higher in the control group in the initial measurements, no statistically significant differentiation was found between the groups. Significant differences were observed more in thickness (*p* = 0.024) and SWE measurements during clenching (*p* = 0.001). The difference in resting readings on the right side suggests a more pronounced effect. Studies by Svensson & Graven-Nielsen and Lobbezoo & Naeije reported that dental pain suppresses masseter muscle activity, particularly during function. This could explain the significant difference observed in our study: significant differences in resting measurements on the right side and only during clenching on the left side [23, 24].

Comparing the first and second measurements, a significant increase in SWE values was observed in the second measurements on the affected side, both at rest and under muscle contraction. This may be due to the regression of protective neuromuscular inhibition caused by SAP inflammation with treatment, and is consistent with previous studies [25, 26]. The observed restoration of muscle thickness and stiffness after root canal treatment serves as a clinical indicator of the stomatognathic system returning to functional homeostasis. This recovery mirrors the quantitative improvements reported in literature following conservative clinical interventions, where muscle stiffness values typically normalize within a range of 2.5 to 4.2 kPa [13, 25]. Furthermore, the reduction in muscle asymmetry post-treatment highlights the effective regression of protective neuromuscular inhibition once the source of periapical inflammation is eliminated [27, 28].

Comparisons made considering the diseased and healthy sides revealed a significant increase in resting SWE, grip thickness, and grip SWE values, particularly in the left masseter muscle, on the diseased side during the second measurement. This indicates that acute inflammation subsided after RCT and that the muscle returned to its normal function with more effective chewing.

It is noteworthy that the gender and age distributions were similar between the case and control groups in our study. Previous studies have shown that gender and age have an effect on masticatory muscles [29]. These close ratios reduce the likelihood that the known effects of gender and age on MMT and elasticity values will create an important difference between the groups.

In this study, the reliability of the measurements was evaluated, and the kappa value was found to be 0.83. This result indicates that the repeatability of USG measurements is at a good level, and the obtained data can be considered methodologically reliable. This level of reliability is consistent with the high intraclass correlation coefficient values (0.84–0.95) reported for trained operators using modern SWE protocols, supporting the method’s success in clinical follow-up [12, 14].

Based on the findings of this study, several recommendations can be made for clinical practice and future research. First, considering the sensitivity of SWE in detecting acute inflammatory changes, this imaging modality could be utilized as an objective ‘biomarker’ to monitor the resolution of periapical infections and the return of masticatory muscle homeostasis [14, 25]. Second, since there is currently a lack of a standardized protocol for masseter USG in endodontic patients, future studies should focus on establishing standardized measurement points and projections (axial vs. coronal) to ensure better intra-operator agreement and comparability between studies [30, 31]. Third, it is recommended that future research investigates the correlation between the severity of periapical lesions (e.g., lesion diameter or pain intensity via VAS scores) and the magnitude of muscle stiffness changes [32, 33]. Lastly, longitudinal studies with follow-up periods exceeding two weeks are warranted to determine whether the masseter muscle fully returns to its pre-inflammatory baseline or if chronic SAP leads to long-term structural adaptations or asymmetry [34].

### Limitations

The limitations of this study include the inability to assess how long before the patient presented to our institution with symptoms of apical periodontitis, absence of long-term follow-up after treatment, and the fact that symptomatic teeth were only considered as posterior teeth and could not be classified.

## Conclusion

SAP leads to a decrease in the thickness and stiffness of the masseter muscle on the affected side; this change can be objectively measured using B-mode USG and SWE. Our findings demonstrate that successful RCT initiates a significant reversal of these biomechanical changes and supports the hypothesis that the adaptation of the masticatory muscles to endodontic pain is reversible. However, the persistence of lower values compared to healthy controls at day 28 suggests that, despite clinical and symptomatic relief, complete physiological restoration of muscle tissue is a progressive process requiring a longer period than the initial healing phase of the periapical tissues.

## Data Availability

It can be obtained from the corresponding author if there is a reasonable reason.
